# Identification of *Spiroplasma* *insolitum* symbionts in *Anopheles gambiae*

**DOI:** 10.12688/wellcomeopenres.12468.1

**Published:** 2017-09-26

**Authors:** Sharon T. Chepkemoi, Enock Mararo, Hellen Butungi, Juan Paredes, Daniel Masiga, Steven P. Sinkins, Jeremy K. Herren

**Affiliations:** 1 Centre for Biotechnology and Bioinformatics (CEBIB), University of Nairobi, Nairobi, Kenya; 2 International Centre of Insect Physiology and Ecology (ICIPE), Kasarani, Nairobi, Kenya; 3 MRC-University of Glasgow Centre for Virus Research, Henry Wellcome Building, Glasgow, UK

**Keywords:** symbiont, malaria, mosquito, Anopheles, Spiroplasma, Plasmodium, vector borne disease

## Abstract

**Background**: Insect symbionts have the potential to block the transmission of vector-borne diseases by their hosts. The advancement of a symbiont-based transmission blocking strategy for malaria requires the identification and study of *Anopheles* symbionts.

**Methods**: High throughput 16S amplicon sequencing was used to profile the bacteria associated with *Anopheles gambiae sensu lato* and identify potential symbionts. The polymerase chain reaction (PCR) with specific primers were subsequently used to monitor symbiont prevalence in field populations, as well as symbiont transmission patterns.

**Results**: We report the discovery of the bacterial symbiont, *Spiroplasma*, in *Anopheles gambiae* in Kenya. We determine that geographically dispersed *Anopheles gambiae *populations in Kenya are infected with *Spiroplasma *at low prevalence levels. Molecular phylogenetics indicates that this *Anopheles gambiae *associated *Spiroplasma *is a member of the *insolitum* clade. We demonstrate that this symbiont is stably maternally transmitted across at least two generations and does not significantly affect the fecundity or egg to adult survival of its host.

**Conclusions**: In diverse insect species, *Spiroplasma* has been found to render their host resistant to infection by pathogens. The identification of a maternally transmitted strain of *Spiroplasma* in *Anopheles gambiae* may therefore open new lines of investigation for the development of symbiont-based strategies for blocking malaria transmission.

## Introduction

Malaria remains a major health problem in many developing countries, particularly in Sub-Saharan Africa ( WHO, 2015). Malaria transmission dynamics are dependent on aspects of the physiology and ecology of their vectors, *Anopheles* mosquitoes *.* Historically, the most successful malaria interventions have been aimed at controlling the vector to break the disease transmission cycle ( Raghavendra *et al*., 2011). The wide distribution of insecticide treated bednets (ITNs) has had a significant impact on reducing the number of malaria cases over the past 15 years, accounting for more than 50% of the malaria deaths averted in this period ( Bhatt *et al*., 2015). However, vector resistance to insecticides used in ITNs is spreading rapidly and there are clear signs of behavioral resistance; mosquitoes that formerly bit indoors are now biting outdoors where nets offer no protection ( Kabbale *et al*., 2013). This may reverse significant reductions in malaria disease burden and therefore new strategies are desperately needed to control mosquito populations or their capacity to transmit parasites. One of the most promising new tools for controlling vector borne diseases involves bacterial symbionts that decrease the vectorial capacity of their insect hosts ( Iturbe-Ormaetxe *et al*., 2011). These symbionts are maintained in host populations through maternal transmission and can spread through insect populations. These features render them potentially a much more sustainable and cost-effective strategy for the control of vector-borne disease transmission than conventional methods ( McGraw & O’Neill, 2013).

In the last decade, there have been many significant advances in the development of symbiont-based strategies for arboviral disease control, primarily centred on the bacterial symbiont, *Wolbachia* ( Jeffries & Walker, 2016). *Wolbachia* can be transinfected into *Ae. aegypti* and *Ae. albopictus* where it blocks the transmission of arboviruses including Dengue, Chikungunya, Yellow Fever and Zika ( Bian *et al*., 2010; Blagrove *et al*., 2013; Blagrove *et al*., 2012; Dutra *et al*., 2016; Ferguson *et al*., 2015; Moreira *et al*., 2009; van den hurk *et al*., 2012; Walker *et al*., 2011; Ye *et al*., 2015). In addition, *Wolbachia*-induced reproductive manipulation (cytoplasmic incompatibility) can drive the rapid spread of this endosymbiont through wild *Ae. aegypti* populations ( Dutra *et al*., 2016; Hoffmann *et al*., 2011). While there is much interest in using a similar strategy to control malaria, there has been limited progress in identifying suitable, maternally transmitted symbionts in *Anopheles* mosquitoes. Numerous studies failed to identify *Wolbachia* from *Anopheles* species ( Ricci *et al*., 2002), and although transinfection of *Anopheles stephensi* has been achieved ( Bian *et al*., 2013), attempts to generate stable transinfected lines of *An. gambiae* have remained unsuccessful. *Wolbachia* has more recently been reported at low frequency, and very low apparent density, from certain field populations of *Anopheles coluzzi and An. gambiae* ( Baldini *et al*., 2014). The natural *Wolbachia – Anopheles gambiae* system reported seems unlikely to have the characteristics required for development as a transmission blocking strategy since it has not been possible to select lines with high density and stable maternal transmission ( Shaw *et al*., 2016).

To advance the prospect of a symbiont-based strategy for malaria control it will be important to continue to identify, generate and study a broad range of *Anopheles* – symbiont systems. Spiroplasmas are members of the Mollicutes, a bacterial group that split from a Gram-positive clostridial lineage of the eubacteria around 600–800 mya and has undergone degenerative evolution. Spiroplasmas are arthropod ‘specialists’ and all known species have some form of interaction with this clade ( Gasparich *et al*., 2004). Members of this genus are functionally diverse, exhibiting a broad array of infection and transmission strategies: they can be pathogens, commensals or mutualists and rely on vertical or horizontal transmission ( Regassa & Gasparich, 2006). In addition, *Spiroplasma* can confer a variety of insect hosts with resistance to a range of eukaryotic parasites, including nematodes, parasitoids and fungal pathogens ( Jaenike *et al*., 2010; Łukasik *et al*., 2013; Xie *et al*., 2010), and they are therefore a good candidate for a symbiont that could be useful for control of *Plasmodium*.

Several *Anopheles* mosquito microbiome surveys have identified *Spiroplasma* from pathogenic clades ( Lindh *et al*., 2005; Segata *et al*., 2016). In this study, we detected the presence of a novel strain of *Spiroplasma* in *Anopheles gambiae* mosquitoes. We sampled *Anopheles gambiae sensu lato* ( *s.l.*) populations from geographically dispersed study sites in Kenya and found that the strain was present at low frequencies across both regions. We have also demonstrated that this *Anopheles* associated *Spiroplasma* is maternally transmitted.

## Methods

### Sampling sites and mosquito collection

Mosquitoes were collected in Karima (0° 41.373'S; 37°19.742’E) and Mbui-Njeru (0° 41.911'S; 37° 20.929'E) villages in Central Kenya region (Mwea) and Kirindo (0° 26'33.1’S 34° 14'58.9’E), Nyawiya (0° 26.7547'S; 34°15.0548'E) and Mageta Island (0° 07.1468'S and 34° 01.018'E) in Western Kenya between April 2016 and July 2017. Karima and Mbui-Njeru villages are located in Mwea, a rice producing region, where rice paddies and associated irrigation canals surrounding the villages provide suitable breeding habitats for *Anopheles* mosquitoes, resulting in very high *Anopheles* mosquito density ( Mwangangi *et al*., 2010). The annual rainfall varies from a maximum of 1,626 mm to a minimum of 356 mm, with an average of 950 mm per year. The average temperatures are 21.3°C (range: 16.0 to 26.5°C) and the relative humidity averages 59.5% (range: 52 to 67%). The Western Kenya region lies within the Kenyan part of the Lake Victoria basin. The main socio-economic activities are small scale fishing and farming. Small sun-lit pools are the main larval habitats for *Anopheles*, mosquito densities are significantly lower than the Mwea region and highly seasonal. The region receives between 250mm and 1200mm of rainfall annually, with the average annual rainfall estimated at 1,100mm. The average temperatures are 22.3° C (range: 15.0 to 28.5°C) and the relative humidity averages 60.5% (range: 51 to 68%). Mosquitoes were collected by manual aspiration in houses and livestock sheds and collection of *Anopheles* mosquito larvae. All collected mosquitoes used in this study were identified as *Anopheles gambiae s.l*. prior to analysis. Mosquito rearing was done in accordance with centre-wide approved standard operating proceedures and occupational health and safety guidelines. The study protocol (NON-KEMRI 545) was approved by the Ethical Review Committee of the Kenya Medical Research Institute (KEMRI/RES/7/3/1). 

### High-throughput 16S rRNA amplicon sequencing

To maximize our chances of detecting potential symbionts we pooled 10 mosquitoes from each location (Central Kenya and Western Kenya). The pools were comprised of DNA from mosquito ovaries (5 mosquito samples) and whole mosquitoes (5 mosquito samples), since endosymbionts are generally at highest density in ovaries but can also be found in high densities in other tissues. The DNA samples were sent to the Research and Testing Laboratory (Lubbock, Texas, USA) for PCR amplification with ‘universal’ 16S rDNA primers ( Lane, 1991; Lane *et al*., 1985), followed by MiSeq illumina sequencing. Samples were amplified in a two-step process that involved 25 µl reaction using Qiagen Hotstart Taq mastermix mix (Qiagen Inc, Valencia, California, USA), 1 µl of each 5 µM primer, and 1 µl of template. Reactions were performed on ABI Veriti thermocyclers (Applied Biosytems, Carlsbad, California). The PCR cycling conditions were 95°C for 5 min, then 25 cycles of 94°C for 30 s, 54°C for 40 s, 72°C for 1 min, followed by one cycle of 72°C for 10 min and 4°C hold. Amplified products were visualized with eGels (Life Technologies, Grand Island, New York, USA). Products were then pooled in equimolar concentrations and each pool was selected using Agencourt AMPure XP (BeckmanCoulter, Indianapolis, Indiana, USA) in ratios of 0.75. The selected pools were then quantified using the Qubit 2.0 fluorometer (Life Technologies, Grand Island, New York, USA) and loaded on a MiSeq Illumina (Illumina, Inc. San Diego, California, USA) 2×300 flow cell at 10 pM. The High-throughput 16S rRNA amplicon sequences reported in this study have been deposited in NCBI under Bioproject number PRJNA399254, Biosample Accession number SAMN07528657 and SAMN07528758.

### *Spiroplasma* screening by PCR and DNA sequencing

DNA was extracted from individual whole mosquitoes and tissues of *An. gambiae s.l.* using a previously described protein-precipitation method ( Herren & Lemaitre, 2011). To screen samples for the presence of *Spiroplasma insolitum* GAMB we used the primers RPOB3044F_ALL and RPOB3284R_INS targeting the 350 bp region of the *rpoB* gene. For molecular phylogenetic analyses, we used the primers SINSFTSZ294F and SINSFTSZ727R (see Supplementary Table 1 for the full list of primers used). The reactions were performed in a 10 µl reaction volume that included 5X HOT FIREPol Blend Master Mix (Solis BioDyne, Tartu, Estonia) and 2 µl of *DNA* template. The cycling conditions included initial enzyme activation at 95°C for 15 min, followed by 35 cycles of denaturation at 95°C for 30 s, annealing at 58°C for 30 s, elongation at 72°C for 30 s, then a hold temperature of 72°C for 7 min. Once samples were found positive for *Spiroplasma,* sequencing of amplicons was performed on a number of samples for validation (Macrogen, Amsterdam, Netherlands). PCR products were cleaned prior to sequencing using the ExoSap-IT purification protocol (USB Corporation, Cleveland, Ohio, USA). The gene sequences generated in this study have been deposited into GenBank under accession numbers MF695842, MF695843, MF695844 and MF695845.

### Molecular phylogenetic analysis

Sequence alignments were performed using Clustal W in Geneious 8.1.9 software ( www.geneious.com, Kearse *et al*., 2012). The trees were constructed by the maximum-likelihood method with a Tamura-Nei model in Geneious 8.1.9 software. Support for tree topology assessed by bootstrap resampling. To determine the phylogenetic position of *Spiroplasma*s identified in this study relative to previously identified *Spiroplasma*s, we compared sequence of 16S rRNA, *rpoB* and *ftsZ* genes. Nucleotide sequences of the other *Spiroplasma* species were derived from GenBank database (accession numbers shown in Figure 3). The length of the compared sequences was 301 bp for 16S rRNA, 210 bp for *rpoB* and 260 bp for *ftsZ*.

### Establishment of iso-female lineages

*Anopheles gambiae* larvae collected from Central Kenya region were reared in the *icipe* mosquito insectary in Mbita, Kenya. Female mosquitoes that successfully reached adult stage were placed in standard 30cm x 30cm x 30cm rearing cages at a density of 30–100 mosquitoes per cage, ensuring a minimum of 30% males. Mosquitoes were then blood fed on *Plasmodium*-uninfected human blood, as previously described ( Gouagna *et al.,* 2003) and allowed to individually oviposit. After oviposition, eggs were counted and each female was screened for the presence of *Spiroplasma*. Adult progeny from infected mothers were counted for egg to adult survival rates and maintained to investigate transmission across multiple generations using the same experimental design. The eggs and adult progeny from some uninfected mothers were also counted to determine egg to adult survival rates. The effects of *Spiroplasma* on female fecundity were determined using female mosquitos collected as larvae from Central Kenya using the same strategy described above. To reduce a potential bias from non-mated females, only broods consisting of more than 10 eggs were used to evaluate fecundity.

### Mitochondrial *DNA* analysis

To determine the diversity of mosquito mt *DNA,* the 655bp *ND5* gene was amplified using the primers described by Besansky ( Besansky *et al*., 1997). Single PCR reactions were performed on the Veriti Thermal Cycler (Applied Biosystems, Carlsbad, CA, USA). PCR cycling conditions were initial denaturation at 95°C for 15 min, followed by 35 cycles of denaturation at 95°C for 30 s, annealing at 55°C for 30 s, elongation at 72°C for 30 s, then a hold temperature of 72°C for 7 min. PCR products were visualized on a 1% agarose gels. The PCR products were purified using ExoSap-IT purification protocol (USB Corporation Cleveland, OH). The DNA Sequences were cleaned and aligned using the MUSCLE algorithm ( Edgar, 2004) in Geneious 8.1.9 software. Minimum spanning haplotype network ( Bandelt *et al*., 1999) was constructed using Popart ( http://popart.otago.ac.nz).

## Results

### *Spiroplasma* sequences isolated from *An. gambiae*

**Figure 1.**
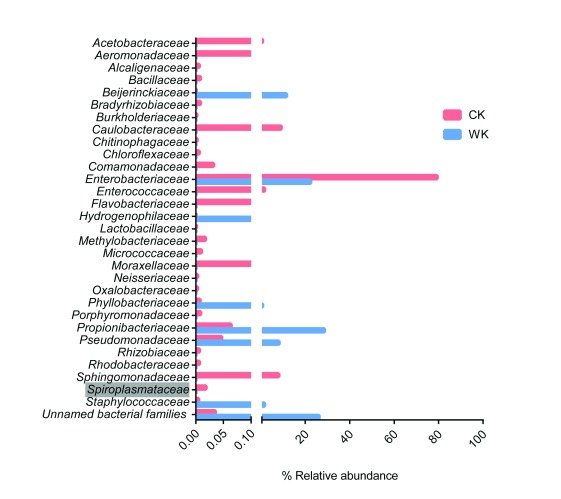
Relative abundance of Bacterial families in *An. gambiae* from Kenya. The relative abundance of bacterial sequences obtained from mosquitoes and mosquitoes from Central (CK) and Western Kenya (WK) Regions as determined by high throughput sequencing of 16s rRNA gene. Notably, 0.02% of the CK reads corresponded to *Spiroplasmataceae*.

Two pools of whole mosquitoes and ovaries were used to generate DNA for High-throughput sequencing of 16S rDNA, which resulted in 195,592 and 18,921 high-quality 16S rRNA sequences, from the Central Kenya pool (CK) and the Western Kenya pool (WK), respectively. *Enterobacteriacea* was most predominant group in CK with approximately 79% of the sequences, whereas both *Propionibacteriaceae* and *Enterobacteriacea* dominated in WK with 26% and 22%, respectively (see Figure 1). In the CK sample, a relatively small fraction of the 16S sequence reads (0.02%) were from *Spiroplasmataceae*. The *Spiroplasma* 16S sequence reads matched the 16S rDNA gene of *Spiroplasma insolitum* strain M55 with 100% identity. *Spiroplasma insolitum* M55 was originally isolated from a flower in Maryland, USA ( Hackett *et al*., 1993).

### *Spiroplasma insolitum* prevalence in field populations of *An. gambiae s.l*

**Figure 2.**
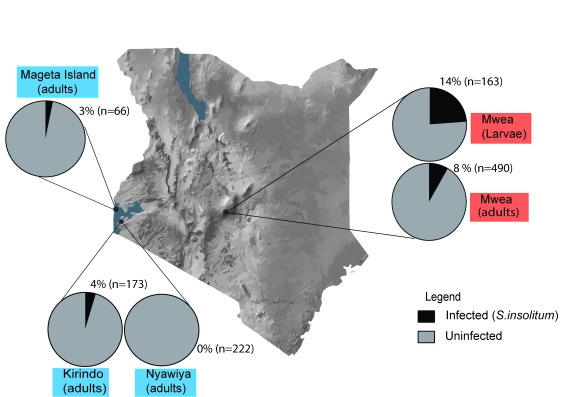
*Spiroplasma* prevalence in *An. gambiae* populations. The prevalence of *Anopheles* associated *Spiroplasma* Western Kenya (blue) and Central Kenya (red). In central Kenya, mosquitoes were collected as both adults and larvae, whereas only adult were collected in Western Kenya. In all cases mosquitoes were screened for *Spiroplasma* as adults. For each site, the prevalence is given as an average of several collections across the course of one rainy season. In central Kenya **,** 8% ( *n*=490) of *An. gambiae s.l* harbored *Spiroplasma*. When collected as larvae, the observed prevalence of infection was higher, at 14% ( *n*=163). Western Kenya sites tended to have a lower prevalence of *Spiroplasma;* Magenta 3% ( *n*=66), Kirindo 4% ( *n*=173) and no infections were found in Nyawiya ( *n*=222).

We developed a set of primers to target the *rpoB* gene of *Spiroplasma insolitum.* These primers were designed based on several *Spiroplasma insolitum rpoB* sequences from previous studies ( Watanabe *et al*., 2013). The specificity of these primers for *Spiroplasma insolitum* was investigated on a panel of diverse *Spiroplasma* species. These primers were then used to determine the population-level prevalence of *Anopheles gambiae* associated *Spiroplasma insolitum* in mosquito samples obtained from Western Kenya (Kirindo, Nyawiya and Mageta Island) and Central Kenya (Mwea), see Figure 2. In all sites, mosquitoes were collected by mouth aspiration in houses across one rainy season (October–December 2016 or April-June 2017). In Mwea, we also collected *Anopheles gambiae* larvae, which were allowed to eclose before being screened for *Spiroplasma* as 21 day old adults. In Mwea **,** approximately 8% ( *n*=490) of *An. gambiae s.l* harbored *Spiroplasma*. When collected as larvae, the rate of infection was higher, at 14% ( *n*=163). In Western Kenya, the prevalence of *Spiroplasma* was generally lower and absent from one site. In Kirindo, the rate of *Spiroplasma* prevalence was 4% ( *n*=173) and in Mageta the prevalence was 3% ( *n*=66), whereas no infections were found in mosquitoes obtained from Nyawiya ( *n*=222).

### Molecular phylogenetic analyses of *Spiroplasma*

**Figure 3.**
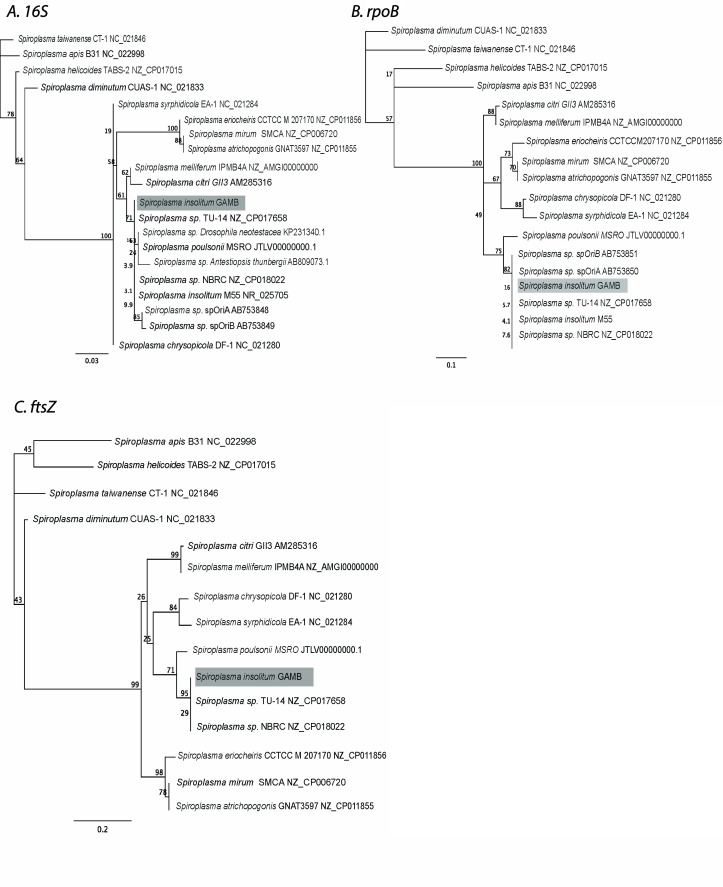
Molecular phylogenetic analysis of *Spiroplasma*. Phylogenies are based on ( **a**) 16s rRNA ( **b**) *rpoB* ( **c**) *ftsZ*. The trees were constructed by the maximum-likelihood method with a Tamura-Nei model using unambiguously aligned sites (total sites are 301, 210, 260 bp, respectively). The numbers above branches indicate a bootstrap value for 1000 replicates. Branch lengths indicate substitutions per site (based on scale bars).

To determine the phylogenetic position of *Anopheles* associated *Spiroplasma insolitum* relative to other members of this clade, and to determine if multiple *Spiroplasma* strains are present in these populations, we developed primers to specifically target and amplify a region of the *Spiroplasma insolitum ftsZ* gene ( Supplementary Table 1). In addition, we sequenced the region of *rpoB* amplified by our *Insolitum*-specific primers. The high-throughput sequencing that we carried out to investigate microbial diversity enabled us to obtain the sequence of a fragment of 16S rDNA. These sequences were used for the construction of phylogenetic trees, which indicate the strain of *Spiroplasma* from *An. gambiae s.l* can be classified into the citri-clade and confirm it clusters with *Spiroplasma insolitum* (see Figure 3). The 16S rDNA fragment sequence was found to be identical to that of three previously described strains of *Spiroplasma insolitum,* M55, TU-14 and NBRC. The sequenced region of the *rpoB* gene from *Anopheles* associated *Spiroplasma* was also found to be identical to M55, TU-14 and NBRC and two strains of *S. insolitum* that are endosymbionts of flower bugs of the genus *Orius, SpOriA/B* ( Watanabe *et al*., 2013). The region we sequenced of the *ftsZ* gene from *Anopheles* associated *Spiroplasma* was identical to M55, TU-14 and NBRC. Notably, *ftsZ* sequence data is not available for *SpOriA/B.* These results indicate that this *Anopheles* associated *Spiroplasma* strain is *Spiroplasma insolitum*, henceforth referred to as *S. insolitum* GAMB. Since all the *S. insolitum* GAMB genes ( *16S rDNA, rpoB* and *ftsZ*) we sequenced were identical, we find no evidence for multiple strains of *S. insolitum* co-existing in the populations of *Anopheles gambiae s.l.* studied.

### *Spiroplasma insolitum* GAMB is maternally transmitted in *Anopheles gambiae s.l* but does not bias sex ratio or affect egg to adult survival

**Figure 4.**
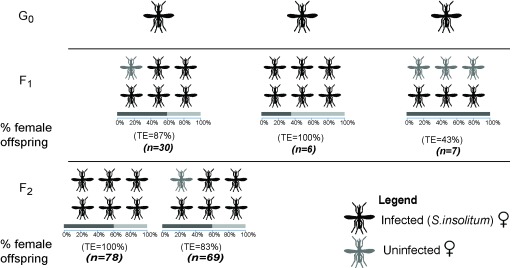
Vertical transmission and sex ratio in *Spiroplasma* infected iso-female lineages. *Anopheles gambiae* mosquitoes collected from Mwea as larvae were used to establish iso-females lineages. Offspring from *Spiroplasma*-infected iso-female lineages were screened for the presence of *Spiroplasma* at adult stage. The number of progeny screened are shown for each female and in the subsequent generation (G _0_, F _1_ and F _2_). At each generation the infection levels and sex ratio were monitored. The bars represent the % sex ratio of the total number of offspring from each iso-female lineage. The presence of between 40%–60% males in all but one family demonstrates that *Spiroplasma* is not a male killer. The observed maternal transmission efficiency (TE), ranged between 43% and 100% with an average of 82.6%.

To determine if *Spiroplasma insolitum* GAMB is maternally transmitted, we collected mosquitoes from the field and established iso-female mosquito lineages. We collected larvae from Mwea (where *Spiroplasma insolitum GAMB* prevalence was highest) and maintained them until they eclosed as G _0_ adults, at which point they were blood fed then allowed to oviposit prior to screening for *Spiroplasma* infection. Three G _0_ females carried *Spiroplasma* and from these, individual F1 female offspring were maintained to enable further screening for *Spiroplasma*. Most F1 did not produce viable offspring; in only one instance we obtained F2 females (see Figure 4). This is not altogether surprising, as field caught *Anopheles gambiae s.l.* are known to perform poorly prior to becoming ‘acclimatized’ to laboratory conditions ( Diop *et al*., 1998). We found that *Spiroplasma insolitum* GAMB is maternally transmitted with very high efficiency, but that transmission efficiency did vary slightly between iso-female lineages. In two cases we observed perfect maternal transmission, whereas the rest had transmission efficiencies between 43% and 87%. The iso-female lineage that produced F2s showed 100% transmission from G0 to F1 and 83% transmission from F1 to F2.

Maternally transmitted symbionts are known to manipulate the sex ratio of their hosts to gain a transmission advantage ( Hurst & Majerus, 1993). To determine if *Spiroplasma insolitum* GAMB affects the sex ratio of *Anopheles* hosts, we monitored the sex ratio of offspring in *Spiroplasma*-infected isofemale lineages. The sex ratio did not differ substantially from the expected 50% female/male in the two lineages producing greater than 10 progeny (see Figure 4), and therefore we conclude that *Spiroplasma insolitum* GAMB is not a male-killer.

**Figure 5.**
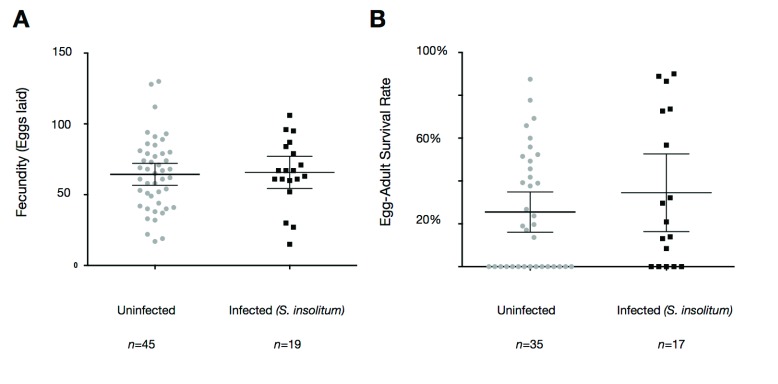
Fecundity and egg to adult survival rates are not significantly affected by *Spiroplasma*. *Anopheles gambiae s.l*. mosquitoes collected as larvae from mwea oviposited individually. The fecundity and the survival of eggs into adulthood was monitored. The fecundity ( **A**) and egg to adult survival rate ( **B**) for *Spiroplasma*-infected mosquitoes was not significantly different from that of uninfected mosquitoes in unpaired t-tests. For fecundity P=0.84, t=1.976, df=62. For egg to adult survival P=0.32, t=1.012, df=50. The data shown is pooled from 3 independent experiments. Shown on the graph are the means with 95% confidence interval.

We also monitored the fecundity and egg to adult survival rate for *Spiroplasma* infected and uninfected iso-female lineages (see Figure 5). We did not observe any significant difference between the fecundity and survival rate of *Spiroplasma* infected and uninfected individuals, indicating that *Spiroplasma insolitum* GAMB is not pathogenic.

### Association between *Spiroplasma insolitum* GAMB and mtDNA haplotypes

**Figure 6.**
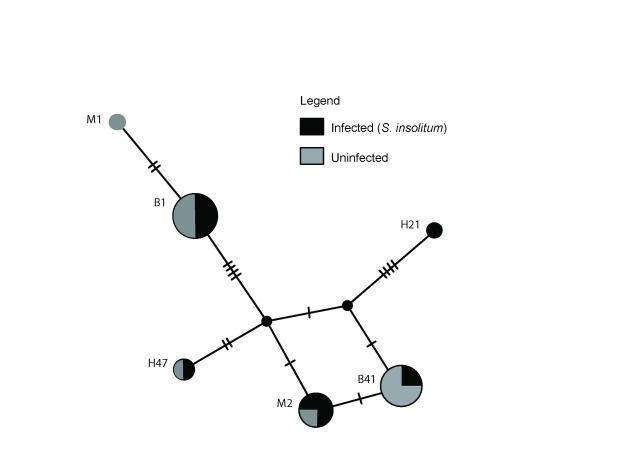
Minimum spanning haplotype network for mitochondrial ND5 gene for Spiroplasma-infected and uninfected mosquitoes from Mwea. The population genetics of *Spiroplasma*-infected and uninfected mosquitoes based on *ND5* mitochondrial DNA loci. Minimum spanning haplotype network reflects loci from 21 mosquitoes, 11 of these were *Spiroplasma*-infected. The circle size corresponds to the haplotype frequencies. The shading reflects the proportion of each haplotype that is *Spiroplasma*-infected. Notably, the majority of haplotypes (B1, H47, M2 and B41) had both *Spiroplasma*-infected and uninfected individuals.

We investigated possible associations between mtDNA haplotypes and *Spiroplasma insolitum* GAMB infection. Symbionts that have recently infected an insect population and are maintained by high efficiency maternal transmission can be expected to be associated with one or a few mitochondrial DNA haplotypes. In contrast, if a symbiont infection is associated with many or most mtDNA haplotypes, this suggests an older infection, paternal transmission or an appreciable level of horizontal transmission in addition to maternal transmission. We sequenced the *ND5* mtDNA gene, which has been widely used for haplotyping *Anopheles gambiae* mosquitoes ( Besansky *et al*., 1997). Of 21 *Anopheles gambiae* specimens collected in Central Kenya (Mwea), 11 were shown to be *Spiroplasma insolitum* GAMB positive based on PCR based screening. We identified a total of 6 haplotypes, four of these are identical to haplotypes reported previously ( Aboud *et al*., 2014; Besansky *et al*., 1997), while two were novel (M1 and M2, see Figure 6). In the 4 most common haplotypes, we found both *Spiroplasma insolitum* GAMB infected and uninfected individuals, suggesting that this symbiont is either an ancient infection or exhibits appreciable levels of horizontal transmission.

## Discussion

We have identified a strain of *Spiroplasma* that is associated with *Anopheles gambiae* mosquitoes in Kenya. This *Spiroplasma* was initially identified from 16S rDNA high throughput sequencing reads from a pool of *Anopheles gambiae* mosquitoes from Central Kenya (Mwea). 16S rDNA sequence revealed that this *Anopheles* associated *Spiroplasma* strain was a member of the citri clade and grouped with *Spiroplasma insolitum*. Most of the known insect endosymbiotic *Spiroplasmas* are found in this clade, for example, *S. poulsonii*, *S. citri* and *S. insolitum* are all species which have been studied as insect endosymbionts ( Haselkorn, 2010; Watanabe *et al*., 2014). It is notable that *Spiroplasma insolitum* GAMB is very closely related to *SpOriA/B*, a strain of *Spiroplasma insolitum* that is an endosymbiont of flower bugs in the genus *Orius* ( Watanabe *et al*., 2014). We are aware of two other studies that identified *Spiroplasma* sequence associated with *Anopheles* mosquitoes. An investigation on midgut bacteria in *Anopheles gambiae* and *Anopheles funestus* from Western Kenya detected 16S sequence corresponding to *Spiroplasma* in *Anopheles funestus* ( Lindh *et al*., 2005) *.* Another study investigated the microbiome of the reproductive tracts of *Anopheles gambiae* and *Anopheles coluzzi* in Burkina Faso and found evidence for the presence of *Spioplasma* in both species ( Segata *et al*., 2016). In both studies, the *Spiroplasma* identified appears to be closely related to *Spiroplasma ixodetus*, which was initially discovered as a pathogen associated with ticks ( Tully *et al*., 1995), and is thus quite different from the *Spiroplasma* identified in our study.

We demonstrate that *Spiroplasma insolitum GAMB* is found at relatively low frequencies in *Anopheles gambiae s.l*. mosquito populations. Frequencies tended to be higher in the Central Kenya Region (Mwea) than in the Western Kenya Region (Mageta, Kirindo and Nyawiya). These two regions have quite different mosquito habitats; in western Kenya most mosquitoes emerge from isolated puddles whereas Mwea (Central Kenya) is a rice growing region where *Anopheles* larvae are abundant in rice paddies and irrigation canals. While both sites experience an increase in mosquito abundance during the rainy season, the difference is less pronounced in Mwea due to year around irrigation providing more permanent larval habitats. We also noted that a higher infection rate was observed in Mwea when we collected larvae instead of adults. A possible explanation for this is that a greater number of these samples had *Spiroplasma* levels that were above the detection limit, or above levels required for the bacteria to be maintained through pupal morphological re-organization into the adult stage. This could be due the favorable laboratory larval and adult rearing conditions, which would likely result in more nutrients available to host and symbiont. Additionally, the mosquitoes collected as larvae (aged to 21 days) were likely to be older than field caught mosquitoes (unknown age). *Spiroplasma* densities in insects are known to significantly increase over the life of the host ( Goto *et al*., 2006; Herren *et al*., 2014) and this could also have caused the observed increase in number of *Spiroplasma* positives.

Since insect associated *Spiroplasma* are known to exhibit a variety of different transmission patterns it was important to determine if *Spiroplasma insolitum* GAMB is maternally transmitted. We established iso-female lineages from infected field collected larvae and demonstrated that maternal transmission does occur with a high level of efficiency. We note that transmission efficiency does appear to vary slightly between the iso-female lineages tested. Two families exhibited very high maternal transmission to F1s, whereas transmission efficiency for the third was about 50%, however the third only generated 6 female offspring. While the spatial localization and mechanistic basis of *Spiroplasma insolitum* GAMB maternal transmission were not investigated, the closely related *Spiroplasma poulsonii* is known to achieve trans-ovarial maternal transmission by subversion of the yolk uptake pathway in *Drosophila melanogaster* ( Herren *et al*., 2013). Other microbial symbionts that persist in the intestinal tract are more likely to achieve maternal transmission by the fecal-oral route. While trans-ovarial maternal transmission tends to be higher efficiency, there are reports of very high efficiency transmission via the fecal-oral route ( Hosokawa *et al*., 2013).

Many maternally transmitted insect symbionts have evolved strategies to bias sex ratio towards females to gain a transmission advantage ( Werren & O’Neill, 1997). The most common manifestation of this is male-killing in which the endosymbiont confers male-specific embryonic lethality ( Hurst & Majerus, 1993). Male killing has been observed in numerous strains of endosymbiotic *Spiroplasma* ( Anbutsu & Fukatsu, 2011). We monitored the sex ratio of the *Spiroplasma*-carrying lineages and found very close to 50% male offspring in the two lineages where more than ten offspring could be examined, suggesting that *Spiroplasma insolitum* GAMB is not a male killer.

A number of *Spiroplasma* are known to be pathogenic to their arthropod hosts ( Clark *et al*., 1985; Nunan *et al*., 2005). By monitoring the fecundity and egg to adult survival rate we determined that *Spiroplasma insolitum* GAMB was not pathogenic. This finding when coupled with its phylogenetic position suggests that *Spiroplasma insolitum* GAMB is likely to either be a commensal or mutualist, although adult fitness assays are also needed. From the standpoint of developing *Spiroplasma insolitum* GAMB as part of a future microbe based transmission blocking strategy this is advantageous, as a pathogenic phenotype could limit the capacity for *Spiroplasma* to spread through the host population.

We did not observe a clear correlation between mt *DNA* haplotype and *Spiroplasma* infection. This could be due to two major possibilities. The first, that *Spiroplasma* infection has been maintained in this species for a very long period of time enabling diversification of mt *DNA* within the infected lineage (as is the case for obligate symbionts, Moran, 2006). A second possibility is that there is significant horizontal transmission of *Spiroplasma* between the *An. gambiae* mosquitoes and/or that paternal as well as maternal transmission occurs, resulting in the wide and almost even distribution of *Spiroplasma* infection between mitochondrial haplotyes. Given that *Spiroplasma insolitum* GAMB is not an obligate symbiont (as it is not found in all *Anopheles gambiae s.l.* individuals), it seems most likely that *Spiroplasma insolitum* GAMB is both horizontally and vertically transmitted. The phylogeny of *Spiroplasma* also suggests a high frequency of horizontal as well as vertical transmission ( Haselkorn *et al*., 2009) and many *Spiroplasma* likely utilize both forms of transmission. From the standpoint of using *Spiroplasma* as a tool for blocking VBD transmission, the prospect of strains being both vertically and horizontally transmitted is of considerable interest and could render them easier to spread through host populations.

We have reported the identification of novel strain of *Spiroplasma* in *Anopheles gambiae s.l.* mosquitoes. Questions that now need to be addressed, once stable infected colonies have been successfully created, include the effects of these *Spiroplasma* on *Plasmodium* transmission, effects on adult lifespan, where it localizes within the mosquito, and mechanisms of vertical and horizontal transmission. *Spiroplasmas* are known to protect a variety of insect hosts from diverse parasites ( Jaenike *et al*., 2010; Łukasik *et al*., 2013; Xie *et al*., 2010) and therefore the discovery of *Spiroplasma insolitum* GAMB could provide a step towards the development of novel malaria control strategies.

## Data availability

The data referenced by this article are under copyright with the following copyright statement: Copyright: © 2017 Chepkemoi ST et al.

Figshare: Identification of *Spiroplasma insolitum* symbionts in *Anopheles gambiae*

Dataset 1 *: Spiroplasma* prevalence in *Anopheles gambiae s.l.* field populations. ( Herren, 2017a)

Dataset DOI: http://dx.doi.org/10.6084/m9.figshare.5384089.v4

Dataset 2: *Spiroplasma* transmission and effects on survival and fecundity. ( Herren, 2017b)

Dataset DOI: http://dx.doi.org/10.6084/m9.figshare.5384101.v3

The High-throughput 16S rRNA amplicon sequences reported in this study have been deposited in NCBI under Bioproject number PRJNA399254, Biosample Accession number SAMN07528657 and SAMN07528758.

The gene sequence data has been deposited in Genbank under accession numbers MF695842, MF695843, MF695844 and MF695845.

## Supplementary material

Supplementary Table 1: Primer sequences used to investigate *Spiroplasma* and host mtDNA haplotype in *Anopheles gambiae s.l.*
