# Supplementary material for: Identification of Spiroplasma insolitum symbionts in Anopheles gambiae
Source: Wellcome Open Res. 2017 Sep 26;2:90. [Version 1] doi: 10.12688/wellcomeopenres.12468.1 (PMC5668936; doi:10.12688/wellcomeopenres.12468.1)
Supplement: Supplementary file 1 [file wellcomeopenres-2-13501-s0000.tgz › 28613c62-15ce-4d5e-9b7b-d51a34eb3b4b.pdf]

Primer name	Sequence	Target Gene / Species	Annealing T_m	Reference
**27F**<br><br>**519R**	AGAGTTTGATCCTGGCTCAG<br><br>GWATTACCGCGGCKGCTG	16S r*RNA*<br><br>(Bacteria)	53°C	(Lane, 1991;<br><br>Lane et al., 1985)
**RPOB3044F_ALL**<br><br>**RPOB3284INS**	ARTHTTACCADTDGAAGATATGCC<br><br>TATCAAATTTTCACCACTACG	*Rpob*<br><br>(*Spiroplasma.insolitum*)	60°C	This study
**SINSFTSZ294F**<br>**SINSFTSZ727R**	TTTTGTTGCCGCTGGAATGG<br>TCGCCTTGTTAGCAGCTTCA	*FtsZ*<br><br>*(Spiroplasma insolitum)*	60°C	This study
**19CL**<br><br>**DMP3A**	CTCCACCAATTACTATAACAG<br><br>AGGATGAGATGGCTTAGGTT	*ND5*<br><br>*(Anopheles gambiae s.l.)*	55°C	(Besansky et al.,<br><br>1997)

Supplementary Table 1: Primer table
