# Supplementary material for: Identification of Spiroplasma insolitum symbionts in Anopheles gambiae
Source: Wellcome Open Res. 2017 Sep 26;2:90. [Version 1] doi: 10.12688/wellcomeopenres.12468.1 (PMC5668936; doi:10.12688/wellcomeopenres.12468.1)
Supplement: Peer review history

# Peer review: Referee response for version 1

**DOI:** 10.21956/wellcomeopenres.13501.r27211

**Published:** 2017-11-02

**Authors:** Brian L. Weiss

**Affiliations:** 1 Department of Epidemiology of Microbial Diseases, Yale School of Public Health, New Haven, CT, USA

In this paper the authors analyze the microbiota of *Anopheles gambiae* mosquitoes from 3 geographically distinct regions of Kenya, with an emphasis on the structure (and to a lesser extent, function) of *Spiroplasma* populations within infected hosts. This analysis is performed by 1) deep sequencing of 16s rRNA sequences from whole mosquitoes and mosquito ovaries, 2) PCR screening for *Spiroplasma* specifically, and 3) observing the effect of *Spiroplasma* infection on female reproductive biology (maternal transmission, fecundity, offspring sex ratio and egg to adult survival). This study, which provides further confirmation that *Spiroplasma* is present in *An. gambiae*, is an important contribution to our collective knowledge of this bacterium’s population structure within this prolific mosquito disease vector.

 I have a few questions/comments regarding the manuscript in its current form:

 The high-throughput experimental design and data representation is ambiguous in places.

- In the Methods, I believe the authors are trying to say that they made 5 samples of ovary and whole organism DNA, each of which contained material from 10 mosquitoes. However, as written in the paper, this is difficult to understand.
- The authors indicate that they used whole mosquitoes “since endosymbionts are generally at highest density in ovaries but can also be found in high densities in other tissues.” If they were interested in characterizing the microbiota found outside of the ovaries, then it might have been a better idea to sequence the 16s rRNA from mosquito carcasses that had the ovaries removed. 
- The Results indicate that only 2 of these 5 pools were sequenced, and only one from each location. This sample size is low (and the Western Kenya pool gave rise to low number of reads). I suspect this analysis was performed as a preliminary indicator of whether or not symbionts are present, and thus whether or not the authors should proceed with further analyses.
- Missing from the Methods is a description of how the 16s data analysis was performed.
- A final ambiguity involves the lack of information regarding microbiota tissue distribution. DNA was extracted from whole mosquitoes and ovaries. Were the whole mosquito and/or ovary specific libraries sequenced seperately? Is the 16s data provided from whole mosquito or ovary samples? If libraries from both sample types were sequenced, it would be interesting if the authors presented the data in a tissue specific manner so as to correlate bacterial diversity and abundance with the distinct tissues used to make the libraries.

For all PCR assays, it would be nice to have more information regarding negative controls. To do so, the authors could have used mosquitoes that they know are *Spiroplasma* negative. I say this because BLAST analysis of the *Rpob* and *FtsZ* primers used in this study revealed that they exhibit very high identity with these genes in other bacteria. Can the authors conclusively rule out non-specific amplification from bacterial members of the microbiota that are not *Spiroplasma*?

I have read this submission. I believe that I have an appropriate level of expertise to confirm that it is of an acceptable scientific standard.

# Peer review: Referee response for version 1

**DOI:** 10.21956/wellcomeopenres.13501.r26401

**Published:** 2017-10-11

**Authors:** Steve J. Perlman

**Affiliations:** 1 Department of Biology, University of Victoria, Victoria, BC, Canada

There is great interest in using host-associated microbes to control insect disease vectors. Maternally transmitted microbial symbionts are especially promising because many have evolved sophisticated strategies to spread through host populations and/or suppress interfere with insect parasites and pathogens. In this study, the authors report the discovery and initial characterization of a strain of Spiroplasma insolitum that infects Anopheles gambiae mosquitoes. It occurs at low frequency in western and central Kenya. The authors establish infected isofemale lines in the lab, rear them for two generations, and show that offspring are infected as well (although transmission efficiency is often low). Mitochondrial sequence analysis suggests that horizontal transmission is pervasive. This is an important contribution to the study of mosquito symbiosis and it will be interesting to characterize this symbiont in greater detail.

 It would be useful to have more information about the establishment and rearing of the iso-female lines (this is not always clear), because it is a bit difficult to interpret the results on transmission to offspring. Were some or all offspring screened as eggs? Were some or all offspring reared individually? If offspring were reared in the same container as larvae, then it is possible that there was horizontal transmission from adult females, and then between larvae. It would be useful to rear infected and uninfected individuals together. If uninfected mosquitoes do not pick up the infection from infected ones, then vertical transmission may predominate. 

 Some other comments:

- It would be useful to have a sentence explaining why Spiroplasma-infected mosquitoes were only kept in the lab for 2 generations.
- The fact that the strain is identical at all genes sequenced in the study to S. insolitum from insects from other orders also suggests predominantly horizontal transmission. It would also be useful to mention more clearly that a number of cultivable/horizontally transmitted Spiroplasma have been isolated from (non-anopheline) mosquitoes.
- In Figure 4, it would be useful to add arrows to indicate which F1 lines are the mothers of the F2.
- It would be useful to have more information about Figure 5. Was transmission to offspring measured in these Spiroplasma-infected females? Which mothers are these (connect to Figure 4? F1?)?
- Why were mosquitoes collected as larvae older than field-caught mosquitoes (second last page)?

I have read this submission. I believe that I have an appropriate level of expertise to confirm that it is of an acceptable scientific standard.

# Peer review: Referee response for version 1

**DOI:** 10.21956/wellcomeopenres.13501.r26402

**Published:** 2017-10-06

**Authors:** Marcelo Jacobs-Lorena, Patricia Hessab Alvarenga

**Affiliations:** 1 Department of Molecular Microbiology and Immunology, Johns Hopkins School of Public Health, Baltimore, MD, USA; 2 Malaria Research Institute, Department of Molecular Microbiology and Immunology, Johns Hopkins Bloomberg School of Public Health (JHSPH), Baltimore, MD, USA

The authors show the relative abundance bacteria families found in *Anopheles gambiae* mosquitoes collected in West Kenya (3 areas) and Central Kenya (2 areas). Thereafter, the authors focus the entire paper on *Spiroplasma, insolitum, *and claim that *this species is different from Spiroplasma already described in African Anopheles mosquitoes.* 

1. The paper should be revised and written more carefully. There are incomplete sentences and also errors in graph (Figure 2, pie graph Mwea does not appear to be 14%).
2. The methodology is poorly described. One example is that the rearing procedures after the mosquitoes were brought from field to laboratory were not described, and in this case are crucial. Another important point: How were the field mosquitoed treated during sample preparation for sequencing? Were the males used to cross with field collected females laboratory sterile mosquitoes, or they were infected too?
3. Moreover the experimental design could be improved considerably:
  - The number of mosquitoes used in different analysis is still too low! Sample size needs to be higher.
  - Although authors say that they had pools with samples of 5 whole mosquitoes and samples of 5 mosquito ovaries, nothing more is done or said about the differences among the two pools. It would be interesting to also include male in the analysis, as well as compare different tissues (including midgut, ovaries, testis and accessory glands, for example).
  - Include sterile laboratory mosquitoes as controls.
  - Authors should compare results using cultivable and culture-independent methods.
  - Experiments performed to verify vertical transmission and sex ratio determination were performed with low number of mosquitoes, two F1 had really low number of offspring  and most of F1 produced no offspring. So, the conclusions to address those questions (maternal transmission and sex ratios) were made based on very few mosquitoes, that came from the low number of parental mosquitoes that were able to produce viable offspring.
  - Fecundity assays performed with low number of mosquitoes. Were those assays made with G0? How those results correlate with the data of Figure 4?
  - In Figure 5, instead of means, this type of data requires medians.
  - It is important to keep in mind that the relative abundance of Spiroplasma,, based on data that the authors had shown (with a low number of pools), is quite low, and the possibility that other bacteria play a role in fecundity and survival rate for example, cannot be ruled out. This was not addressed or discussed.
4. In my opinion data provided and sample size are not enough to support the conclusions.
5. Not clear why authors focused on *Spiroplasma*, that has low relative abundance and prevalence in the areas and claim they might be good symbiont to protect mosquitoes from *Plasmodium*. A lot needs to be done and better addressed.
6. Discussion is still poor.

We have read this submission. We believe that we have an appropriate level of expertise to state that we do not consider it to be of an acceptable scientific standard, for reasons outlined above.
